# Knowledge of rabies among rural community in Chengalpet district, India

**DOI:** 10.6026/97320630018155

**Published:** 2022-03-31

**Authors:** Kamini Bharani, Karthikeyan Ramachandran, Vaishnavi Kommisetty, Krishna Prasanth Baalann

**Affiliations:** 1 Department of Community Medicine, Sri Muthukumaran Medical College and Research Institute, Chennai, India; 2 Department of Physical Medicine & Rehabilitation, Sree Balaji Medical College & Hospital, Bharath Institute of Higher Education & Research, Chennai, India; 3 Department of Community Medicine, Meenakshi Medical College & Research Institute, Meenakshi Academy of Higher Education & Research, Chennai, India; 4 Department of Community Medicine, Sree Balaji Medical College & Hospital, Bharath Institute of Higher Education & Research, Chennai, India

**Keywords:** Rabies, Knowledge, Post exposure prophylaxis

## Abstract

The objective of the present study is to assess the knowledge about rabies among the general population of a rural area in Chengalpattu district of Tamilnadu. A cross-sectional study was conducted among 361 participants belonging to Paranur village of Chengalpattu district. A pre-tested questionnaire was utilized to gather information on socio-demographic factors, knowledge about rabies, treatment-seeking behavior, and anti-rabies vaccine use among participants with history of dog bite. Among the 361 participants, only 49.5 % were aware of local wound-management procedures, despite the fact that 68% had sufficient knowledge about rabies. The present study highlights. significant association between knowledge regarding rabies with demographic variables such as gender, age, education and occupation Although more than half of the study population had adequate knowledge on rabies, one-fourth of them had no knowledge on first-aid treatments or vaccines at the time of dog bite. This study highlights the need to promote knowledge regarding wound care and post-exposure prophylaxis at the event of a dog bite.

## Background:

Rabies is one of the world's most neglected tropical illnesses, affecting primarily poor and vulnerable people in both rural and urban areas [1-36]. Rabies is a preventable disease caused by a virus which is usually lethal once clinical symptoms appear. The rabies virus is primarily transmitted to people by domestic dogs, [14] although it can also be transmitted by other domestic pets and wild animals [2,30,35]. It is contracted by being exposed to saliva of the rabid animal through bites or scratches [20,21]. Rabies is found across all continents except Antarctica, with Asia and Africa accounting for almost 95 % mortality [22, 23,24,32&^46^]. The World Health Organization states that Rabies claims the lives of an estimated 59,000 people each year globally, out of which 20,000 are in India, accounting for the largest number of rabies deaths globally [19]. Most of these deaths occur primarily among children in rural or marginalized populations [1]. Rabies related deaths are seldom reported globally, and it is believed that children aged 5 to 14 are the most common victims [4, 33]. Globally individuals requiring post-bite immunization amounts to over 29 million people, every year. This knowledge aids us in averting hundreds of thousands of rabies deaths each year. [8] Along with research and advancements on vaccines [12,26,38,40,47] and vaccination strategies for animals [17,18,34,41,42, 44,50] those transmit rabies, there are potent human rabies vaccines and immunoglobulins for effective management of rabies. But there seems to be a problem in availability and affordability of these for the individuals nursing a dog bite. Simple countermeasures, such as cleansing bite wounds with water and soap, can make a significant difference in averting rabies related mortality among vulnerable populations. Adequate knowledge on demographics of domestic dogs and their vaccination with awareness among dog owners about rabies is an essential factor for controlling human rabies infection. [16,27, 29,48,49] Health education to both children and adults, regarding rabies and the first aid subsequent to dog bite, is an important component of rabies prevention program. [13,15,25,28,31, 43,45] This indirectly reduces the incidence of human rabies and well as the cost of treating dog bites which is a key stumbling block in the fight against rabies. [3] Therefore, it is of interest to document knowledge on rabies among rural people at Chengalpet district, Tamilnadu, India.

## Materials and Methods:

### Sample size:

The sample size for this study was estimated by using the data from a study on Knowledge on rabies among the rural population of India. The required sample size was estimated to be 361 individuals belonging to Paranur village, which has population of 6462 in 1682 households. The sample size was estimated with confidence level of 98 % and 2% error.

### Survey method:

In the village of Paranur of Chengalpet district, Tamilnadu, a cross-sectional household survey was conducted. According to the census of India, the village has a population of 6462 in 1682 households. There had been no previous awareness campaigns on rabies or dog population control measures in the area. Six clusters were framed among the population. The village head was asked to suggest houses from each cluster to prevent respondents from introducing bias. The questionnaire survey included a total of 50 households per each cluster. Each family's head was approached to fill up the questionnaire. Before starting the survey, a document describing informed consent was read to the respondents in the local language (Tamil) and verbal consent was received. If any of the households declined to take part in the survey, the study was forwarded on to the next household.

### Questionnaire design:

The questionnaire included closed questions about:

(1) Household information to determine socioeconomic status and resident description, such as age, gender, education, occupation etc.

(2) Rabies knowledge, attitudes, and practices (a total of 10 questions about knowledge).

The interviewer read the questions to the responders in their native language.

### Data management and analysis:

Answers to the questions were entered in the MS EXCEL and the analysis was done using SPSS version 24. Demographic data was given in frequency and percentage. A chi-square test was used to assess the relationship between this outcome variable and several demographic and associated factors. In the final model, variables which had a statistical significance p<0.05 were reported.

## Results:

Table 1(see PDF) shows the demographic characteristics of the individuals. The age groups were given in mean and standard deviation as 44.76 (24.484), respectively. Most of the study participants were male (60%) and only 47% of the study population was working. Almost half (50%) of the study participants had school level education and 49% of them hailed from middle income group.

## Respondents Knowledge regarding Rabies (Table 2 - see PDF):

Majority of the study participants (55%) had heard of the term "rabies". 61% were aware that rabies could spread through dog bites, 55% replied that domestic animals could transmit rabies. 65% knew the local treatment for animal bite and 68% opinioned that rabies can be prevented. Only a mere 33% of them knew that animal bite should be washed with water and soap. 65% of them said rabies can be prevented by vaccinating dogs. Over all, only 60% of them had desirable knowledge on rabies transmission, prevention and control.

It is inferred from Table 3(see PDF) that variables determining knowledge such as "heard the term rabies" and animal bite can transmit rabies had a statistical significant association with gender. All other knowledge variables had no association with gender.

## Discussion:

It is reported that majority of study participants had only heard about the term "rabies". Nearly half of the respondents had knowledge about rabies prevention and control. While comparing with other demographic variable with knowledge there was no association observed in this study such as a lack of knowledge regarding importance of wound washing, the risk of transmission of rabies infection from species other than dogs which was similar with the study done by Samanta M et al. [4]. In contrast, another study done in rural Pune revealed a lower prevalence (less than 10%) of awareness regarding dog bite [5]. This difference in knowledge may be due to the fact that a greater number of participants had no formal education. Regarding knowledge about fatal nature of rabies, our study showed that about 60% believed that Rabies was curable. However, studies done in other rural parts of the country like Pune, Gujarat, New Delhi and a study done in urban Pondicherry have revealed that almost 90% had knowledge about the fatality from rabies which is higher than the findings observed in the current study [6,7]. In our findings, half of the study participants were unaware of the preventive measures for rabies. Similar findings (52.1%) were also found in a study done in rural community of Pune [4] and study conducted in rural areas of eastern India [8]. Though rabies control program was implemented in India, there was a dire need to create awareness and periodic surveillance among the community in rural parts of the country where dog bites are more prevalent [39,51]. Knowledge regarding anti rabies vaccine availability was found to be very less in this rural population. Similar results were observed in studies conducted by Mantong Zhu in China [9] which was contrast to study conducted in urban Pondicherry. Conversely, studies done in rural community of Gujarat (86%) and Bangladesh (70%) showed lesser awareness as compared to our study findings [10]. The present study highlights the association between knowledge regarding rabies with demographic variables such as gender, age, education and occupation which was contrary in another study where rabies prevention and control in four rural areas of Sri Lanka [11]. However, other knowledge-based variables such as questions like washing the site of bite with water and soap, and vaccination for rabies prevention, had a strong association with the participant’s occupation. Before approaching a health care facility, simple measures such as washing the bite wound with water and soap are part of post exposure prophylaxis and play an important role in healing of tissue. However, the current study highlights a knowledge gap in the rural areas about the course of action to be taken after dog bite which needs to be addressed through regular targeted awareness program, as presented in the study of Matibag GC et al. [28]. Also, this study highlights the need for further studies on rabies in various parts of the world, with varied socio economic, educational and cultural backgrounds [25,52]. The findings of this study thereby throw light on significant factors influencing rabies awareness in rural areas of the Chengalpattu district which should be focused to enhance the health-seeking behavior and rabies control measures.

## Conclusion:

People living in rural area have inadequate knowledge on rabies and its prevention. Considering the wide variation in knowledge about rabies prevention and control in the community, there is a dire requirement to raise awareness about this disease and post-exposure prophylaxis which are the only way to eliminate this otherwise fatal disease.
